# Giant Panda Maternal Care: A Test of the Experience Constraint Hypothesis

**DOI:** 10.1038/srep27509

**Published:** 2016-06-07

**Authors:** Rebecca J. Snyder, Bonnie M. Perdue, Zhihe Zhang, Terry L. Maple, Benjamin D. Charlton

**Affiliations:** 1 Zoo Atlanta 800 Cherokee Avenue SE Atlanta, GA 30315, U.S.A.; 2 Oklahoma City Zoo and Botanical Garden 2101 NE 50th Street Oklahoma City, OK 73111, U.S.A.; 3 Agnes Scott College 141 E. College Avenue Decatur, GA 30030, U.S.A.; 4 Chengdu Research Base for Giant Panda Breeding 1375 Xiongmao Avenue, Chenghua Chengdu, Sichuan, People’s Republic of China 610081; 5 Jacksonville Zoo and Gardens 370 Zoo Parkway Jacksonville, FL 32218, U.S.A.; 6 School of Biology and Environmental Science, University College Dublin (UCD), Belfield, Dublin 4, Ireland

## Abstract

The body condition constraint and the experience condition constraint hypotheses have both been proposed to account for differences in reproductive success between multiparous (experienced) and primiparous (first-time) mothers. However, because primiparous mothers are typically characterized by both inferior body condition and lack of experience when compared to multiparous mothers, interpreting experience related differences in maternal care as support for either the body condition constraint hypothesis or the experience constraint hypothesis is extremely difficult. Here, we examined maternal behaviour in captive giant pandas, allowing us to simultaneously control for body condition and provide a rigorous test of the experience constraint hypothesis in this endangered animal. We found that multiparous mothers spent more time engaged in key maternal behaviours (nursing, grooming, and holding cubs) and had significantly less vocal cubs than primiparous mothers. This study provides the first evidence supporting the experience constraint hypothesis in the order Carnivora, and may have utility for captive breeding programs in which it is important to monitor the welfare of this species’ highly altricial cubs, whose survival is almost entirely dependent on receiving adequate maternal care during the first few weeks of life.

In mammalian and avian species in which females reproduce more than once in their lifetimes, reproductive success generally increases early in life, and then subsequently remains constant until reproductive senescence^1^,^2^,^3^,^4^. The increase in reproductive success early in life may emerge because mothers undergo neural^5^,^6^, hormonal^7^, physiological^8^, and behavioural changes^2^,^9^ particularly from their first pregnancy and birth to the next. Consistent with this, in a number of mammal species primiparous mothers (i.e., those having given birth to their first offspring) have often been found to be more neglectful (sheep, *Ovies aries*:^10^,^11^; bottlenose dolphins:^12^; cows, *Bos taurus*:^13^; prairie voles, *Microtus ochrogaster*:^9^; gray mouse lemurs, *Microcebus murinus*:^14^; rhesus and Japanese macaques, *Macaca mulata* and *Macaca fuscata*^15^,^16^; Western lowland gorilla*s, Gorilla gorilla gorilla*^17^,^18^; chimpanzees, *Pan troglodytes*:^19^) and/or experience lower reproductive success than experienced or multiparous mothers (prairie voles, *Microtus ochrogaster*:^9^; cape and Columbian ground squirrels, *Xerus inauris* and *Urocitellus columbianus*:^1^,^4^; rhesus macaques, *Macaca mulata*:^20^; hamadryas baboons, *Papio hamadryas*:^21^; primates:^22^; polar bears, *Ursus maritimus*:^23^; brown bears, *Ursus arctos*:^24^).

Constraint hypotheses predict that differences in reproductive success emerge as a result of reproductive performance being constrained in primiparous mothers by various factors^1^,^3^,^4^,^8^. Specifically, the *body condition constraint hypothesis* proposes that primiparous mothers have lower reproductive output because of lower body mass or other deficits in bodily condition^1^. First time mothers are often not fully grown at the time of first parturition which results in a tradeoff between reproduction and their own growth/survival^8^. Alternatively, the *experience constraint hypothesis* proposes that primiparous individuals have lower reproductive performance because they lack adequate reproductive and/or maintenance experience (e.g., foraging skills)^1^,^4^,^8^,^9^. However, primiparous mothers are typically characterized by both inferior body condition and lack of experience when compared to multiparous mothers^1^,^25^, and hence, interpreting experience related differences in maternal care as support for either the body condition constraint hypothesis or the experience constraint hypothesis is extremely difficult.

Giant panda (*Ailuropoda melanoleuca*) females are considered reproductively mature at 4.5–5.5 years of age^26^,^27^,^28^ and typically undergo one estrous period per year, unless they have a dependent cub^26^,^28^. Based on captive data, twinning occurs in nearly half of all litters, but mothers typically abandon one neonate^28^. At birth, giant panda cubs are highly altricial and mothers support cubs on their bodies nearly constantly for the first few weeks after parturition^28^. Cubs are also extremely vocal during the first few weeks of their life^26^,^28^ which suggests that vocal signals are important for communicating the cub’s need to the mother at a time when it is completely dependent on maternal care^26^,^28^. In addition, the altricial state of the neonate makes a denning period necessary for cub survival^28^. Denning typically lasts around three to four months, and mothers may move the cub to multiple den sites^28^. Following the denning period, cubs will typically remain with their mothers until the age of 1.5–2.5 years^26^,^29^. Some researchers have described mother-infant interactions during this denning period^28^,^30^, but substantial quantitative data from multiple mothers are lacking for this critical period of maternal care.

Moreover, because female giant pandas experience little weight change during pregnancy and their fetuses are extremely small (90–130 g) at birth^26^,^31^, primiparous and multiparous females would not be expected to experience significant changes in body condition during pregnancy. Accordingly, by studying captive giant pandas that are consistently provisioned with high quality food and are subject to regular health checks, the effect of body condition on primiparous and multiparous mothers is mostly controlled, and it is possible to provide clear support for or against the *experience constraint hypothesis* in this species. If multiparous giant pandas exhibit more proficient maternal care, this would support the hypothesis that a lack of experience, rather than body condition, constrains maternal behaviour in primiparous giant panda mothers.

Here we provide long-term data on mother-infant interactions gathered over a period of ten years for eight mothers and 21 giant panda cubs born in captivity. By examining this dataset, we were able to quantitatively describe the pattern of maternal behaviour during the denning period, and investigate the influence of previous experience on maternal care. Based on the experience constraint hypothesis, we predicted that experienced (i.e., multiparous) giant panda mothers would provide more proficient maternal care to offspring than inexperienced (i.e., primiparous) mothers. Specifically, we predicted that experienced mothers would spend more time nursing, grooming, and holding their cubs, and also have less vocal cubs than inexperienced mothers due to their greater ability to provide adequate maternal care that satisfies the immediate needs of the offspring. In addition, because anogenital stimulation by the mother is required for altricial young to urinate and defecate^5^,^29^, we also predicted that experienced giant panda mothers would spend more time licking the anogenital region of their cubs.

## Results

**Figure 1:**
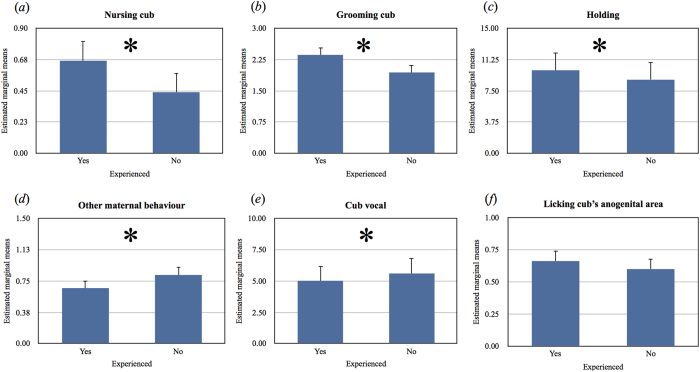
Estimated marginal means ± s.e. generated by GLMMs fitted with a Poisson probability distribution for experienced and inexperienced captive giant panda mothers for nursing behaviour (**a**) cub grooming (**b**) holding the cub (**c**) other maternal behaviour (**d**) cub vocal behaviour (**e**) and licking the cub’s anogenital area (**f**), *p < 0.05.

To test our hypotheses we used generalized linear mixed models (GLMMs) in which the mother’s identity was entered as a random factor and her prior experience of raising offspring or not (experienced versus inexperienced) was entered as a fixed factor categorical variable. There was no significant difference between the mean body weight of inexperienced and experienced female giant pandas (*F*_1, 11_ = 0.687, *P* = 0.425), confirming that the primiparous and multiparous females in our study did not differ greatly in size, and hence, were very unlikely to have differing energetic constraints that might have influenced their ability to provide maternal care. Subsequent GLMMs revealed that multiparous mothers spent significantly more time nursing their cubs (*F*_1, 2000_ = 11.361, *P* = 0.001), grooming their cubs (*F*_1, 2000_ = 10.605, *P* = 0.001), and holding their cubs (*F*_1, 2000_ = 19.060, *P* < 0.001) but less time engaged in other maternal behaviour (*F*_1, 2000_ = 2.580, *P* = 0.108) compared to primiparous mothers (Fig. 1a–d, respectively). In addition, the cubs of multiparous mothers were significantly less vocal than the cubs of primiparous mothers (*F*_1, 2000_ = 8.945, *P* = 0.003) (Fig. 1e). In contrast, no significant difference in the amount of time primiparous versus multiparous mothers spent licking their cubs’ anogenital areas was observed (*F*_1, 2000_ = 1.061, *P* = 0.303) (Fig. 1f).

The mean body weight of each giant panda mother for the year in which the behavioural observations were conducted, the cub’s age, and whether mothers had twins or not were also entered into our GLMM analysis to control for these factors. We found that heavier female giant pandas spent less time holding their cubs (*F*_1, 2000_ = 113.513, *P* < 0.001) and had less vocal cubs (*F*_1, 2000_ = 96.032, *P* < 0.001), however, body weight did not significantly affect the amount of time spent nursing (*F*_1, 2000_ = 1.363 *P* = 0.243), grooming (*F*_1, 2000_ = 0.261 *P* = 0.609), licking the cub’s anogenital region (*F*_1, 2000_ = 0.339 *P* = 0.560), or engaged in other maternal behaviour (*F*_1, 2000_ = 0.792 *P* = 0.374). In addition, younger cubs were more vocal than older cubs (*F*_1, 2000_ = 1190.341, *P* < 0.001), and giant panda mothers spent significantly more time grooming (*F*_1, 2000_ = 47.328 *P* < 0.001), holding (*F*_1, 2000_ = 8963.549 *P* < 0.001), licking the anogenital region (*F*_1, 2000_ = 4,533 *P* = 0.033), and less time nursing (*F*_1, 2000_ = 17.110 *P* < 0.001) younger cubs. In contrast, no effect of the cub’s age on other maternal behaviour was detected (*F*_1, 2000_ = 1.471 *P* = 0.225). Finally, although the amount of time engaged in anogenital licking (*F*_1, 2000_ = 0.010 *P* = 0.919) and holding (*F*_1, 2000_ = 0.759 *P* = 0.384) cubs did not differ according to whether mothers had twins or singletons, mothers with twins had more vocal cubs (*F*_1, 2000_ = 4.428, *P* = 0.035), spent more time nursing (*F*_1, 2000_ = 137.222 *P* < 0.001) and engaged in other maternal behaviour (*F*_1, 2000_ = 6.344 *P* < 0.012), and less time grooming cubs (*F*_1, 2000_ = 22.150 *P* < 0.001) than those with singletons.

## Discussion

Our results provide clear evidence that multiparous (experienced) giant panda mothers provide more maternal care for their highly altricial offspring than primiparous (first time) giant panda mothers. Specifically, we found that multiparous giant panda mothers spent more time nursing, grooming, and holding their cubs than primiparous mothers. Since body condition was largely controlled for in this study on well-provisioned captive animals, our findings in giant pandas provide the first strong support for the experience constraint hypothesis of maternal care in the order Carnivora.

The behaviours performed more by multiparous mothers (i.e., grooming, holding and nursing) are especially critical for survival of giant panda neonates, which even within ursids are the smallest compared to the weight of the mother of all placental mammals^26^,^28^. Giant panda mothers are thought to help offspring thermoregulate by holding them close to their body during the first few weeks after birth^26^,^28^. Consequently, we suggest that multiparous female giant pandas, which spend more time holding cubs, are better at helping their cubs maintain a stable body temperature than primiparous mothers. We also expected that multiparous mothers would spend more time licking their cubs’ anogenital areas compared to primiparous mothers because anogenital stimulation by the mother is necessary for altricial young to urinate and defecate^5^,^28^, however, this prediction was not supported by the results. Nonetheless, multiparous mothers did spend more time grooming cubs, which is also likely to be important for keeping the infant clean during this critical early stage of its development.

Furthermore, giant panda mothers actively hold and position their cubs for nursing which requires considerable dexterity^28^, as opposed to black and brown bears which give birth during hibernation and do not hold their cubs to facilitate suckling^28^. Experienced giant panda mothers are therefore more likely to be proficient at positioning their cubs near nipples and at adjusting their own body position to allow cubs easier access to nipples, and thus can facilitate longer nursing bouts, possibly resulting in faster growth rates. Based on the results of previous studies^24^,^32^ and our finding that experienced mothers spend more time nursing, we would expect that multiparous panda mothers would have cubs with faster growth rates. We were not able provide evidence for this in the current study, because birth weights and subsequent cub weights to measure growth rates were not available. Thus, future studies that examine the relationship between giant panda cub growth rates and maternal care are certainly warranted.

We also found that multiparous giant panda mothers had less vocal cubs than primiparous mothers. Giant panda cubs are highly vocal in the first few weeks of life^26^,^28^ and cub calls have been shown to contain information about the caller’s arousal state^33^. Thus, giant panda cub vocalisations are believed to be critical for communicating the cub’s needs to the mother and eliciting maternal attention and care^26^,^34^. We expected that experienced mothers would be more responsive to cub vocalisations, and thus better able to sooth and quiet their cubs and our findings support this. In addition, during our observations we noted that giant panda mothers normally respond to vocalizing cubs by repositioning them, in order to facilitate nursing or grooming, or to cover more of the cub’s body. Therefore, because the behavioural category other maternal behaviour includes repositioning the cub, higher cub vocalisation rates may also explain why primiparous mothers spent significantly more time engaged in other maternal behaviour.

It is important to note that all the mothers in our study provided adequate species-typical maternal care for their offspring, with all the cubs surviving into adulthood. Captive giant pandas sometimes fail to care for their offspring^31^,^35^. For instance, some mothers respond fearfully to their neonate immediately after giving birth and fail to pick the cub up (R. Snyder, personal observation), particularly first time mothers^36^. However, some females also continue to provide inadequate maternal care after giving birth multiple times^36^, either in the form of early rejection or improper holding, positioning, and grooming. Since giant panda neonates are highly altricial and susceptible to hypothermia^26^ animal care staff are quick to remove cubs that are not picked up by the mother within a minute or two after birth, closely monitor mother-cub behaviour throughout the denning phase, and promptly remove the cub from the mother if they suspect that it is not receiving adequate care. Given the very limited time that mothers displaying inadequate care have with their cubs, we chose to focus our study on mothers providing adequate care. Therefore, we suggest that the experience effect observed in the current study is particularly robust because we limited our dataset to competent mothers (i.e. those that were able to successfully rear offspring) and still revealed significant differences between experienced and inexperienced mothers. In addition, we suggest that our findings may have utility for captive breeding programs. For example, conservation managers could monitor first time giant panda mothers more closely than experienced individuals, allowing them to ensure that cubs receive adequate maternal care during the first few weeks of their life, when it is likely to have a critical impact on offspring survival.

Although the results of this study provide strong support for the experience constraint hypothesis, we cannot rule out the possibility that maternal restraint also operates on giant panda maternal behaviour in the wild. The maternal restraint hypothesis predicts that primiparous mothers limit their investment in their first reproductive effort to avoid risking their survival and future reproduction^1^,^4^,^8^. The captive female giant pandas in the current study did not face a risk to their own survival by spending time intensively caring for their cubs, unlike wild giant panda mothers that need to balance the energy demands of remaining in the den to care for their vulnerable young versus spending time away foraging for food in order to survive. In terms of maternal restraint, it is also important to note the distinction between maternal investment and maternal care. Maternal care refers to behaviour that benefits the offspring at no cost to the mother, such as sharing a sleeping site^37^, whereas maternal investment implies that the benefit conferred to the offspring results in a cost to the mother, such as delaying or decreasing the probability of future reproduction^38^.

Demonstrating that maternal behaviour results in a cost to the mother is sometimes challenging, but in bears that do not hibernate, such as giant pandas and sun bears, time spent in a den actively caring for offspring most likely reflects a form of investment^39^. Furthermore, giant pandas feed almost exclusively on bamboo, which is low in nutrients and requires extensive foraging and processing time^26^. This prevents giant pandas from increasing consumption and storing fat prior to giving birth^26^. Additionally, they undergo a fasting period following parturition^28^,^29^. As a consequence, giving birth and providing the necessary care to keep the infant alive is likely to have an immediate and direct cost to the mother in the wild, making it possible that free-ranging individuals also limit their maternal investment to ensure that they survive and reproduce in future years. By using captive giant pandas in the current study we were able to remove the energetic costs of foraging for food and document the reproductive histories of our study animals, thereby allowing us to present the first strong support that the experience constraint hypothesis accounts for differences in maternal behaviour in the order Carnivora. Our study also demonstrates the importance of using captive animals as research subjects when investigating how prior experience affects maternal behaviour, particularly in endangered species like the giant panda in which effective *ex situ* breeding is essential for conservation.

## Methods

### Subjects and Housing

The subjects were eight female giant pandas ranging in age from 5 to 16 years. All cubs included in this study survived into adulthood and four mothers were studied in two or more years/litters. All the giant pandas were born in captivity and kept in captivity throughout this study. Six mothers (i.e., BingBing, ChengCheng, ChengJi, ErYatou, MeiMei, and YaYa) were housed at the CRBGPB, Sichuan, People’s Republic of China. One mother, QingQing, was housed at the Chengdu Zoo, Sichuan, P.R. China for one study year, and at the CRBGPB for a second study year. One mother, LunLun, was housed at Zoo Atlanta, Georgia, United States of America (Table 1 lists the locations of each female giant panda in the study).

During this study, subjects housed at the CRBGPB were kept in indoor enclosures with concrete floors, concrete walls on one or two sides and walls of steel bars on two or three sides. The enclosures measured 12 m^2^. The subject housed at Chengdu Zoo was kept in an indoor enclosure of the same construction that measured 11 m^2^. All subjects housed in China remained in these enclosures throughout the study. They were shifted into an adjacent indoor enclosure of similar size for a few minutes daily as needed for cleaning. These subjects were provided with grass mats for bedding and used the matted corner of the enclosure as the nest where they kept their cubs. The female giant panda housed at Zoo Atlanta was kept in a 10 m^2^ indoor enclosure with cement floor, three cement walls, and one steel mesh wall for the first two weeks after her cubs were born. This enclosure included a 52 × 40 cm nest box in one corner with hay bedding. She kept her cub in this nest box throughout the study. Two weeks after giving birth, this subject was given access to an adjacent enclosure of the same size and construction.

Although it is normal for giant panda mothers to fast for a few days to as many as 25 days after giving birth^28^,^29^, all mothers in this study had bamboo and/or bamboo shoots and water available in their enclosures from the time they gave birth throughout the entire study. The farthest distance any mother had to travel to obtain food or water was two meters from the nest where she kept her cub. All mothers were also offered a concentrated food. At the Chinese institutions this was rice, corn, and soybean flour-based bread, which was made by staff at these institutions. The concentrated food was provided five to ten times per day depending on the mother’s appetite. At Zoo Atlanta this was a commercially available soybean based high fiber biscuit, which was provided eight times per 24 hour period. The amount of concentrated food offered varied according to the mother’s appetite and the cub’s age.

The procedures used in the research did not affect the housing, diet, or management of the animals and comply with the law of the People’s Republic of China. The director of CRBGPB and Zoo Atlanta’s Scientific Review Committee approved the research. All procedures were performed in accordance with the guidelines established by CRBGPB and Zoo Atlanta’s Scientific Review Committee.

### Data Collection

Behavioural data collection began as soon after birth as possible and continued until cubs were 91 days old. For BingBing, ChengCheng, ErYatou, LunLun, and YaYa, data collection began within the first day after the cub or cubs were born. For ChengJi, data collection started when her twins were two days old. Data collection for Mei Mei began when her twins were three days old. For QingQing, data collection in 2000 began when her cub was one day old and in 2001 it began when the cubs were two and three days old. Observations were made between 0700−1700. Table 1 lists the year the observations were conducted, the birth history, mean body weight, litter size, captive location and number of hours of data collected for each mother.

Female giant pandas that had twins only had one cub with them at any given time. The Chengdu institutions use a swapping procedure for twins whereby the two cubs are alternated between their mother and an incubator. This procedure is commonly used by Chinese institutions to ensure that each cub receives adequate care from the mother, and to reduce the chance that the mother will abandon or accidentally injure one of the cubs^40^. The schedule for alternating cubs varies by mother, cub age, and cub condition. We tried to collect an equal amount of data on each mother-twin dyad, but this was not always possible because of the swapping schedule.

Thirty-minute focal observation sessions were used to record behavioural information for each mother. Instantaneous sampling at one-minute intervals^41^,^42^ was used to record the following maternal behaviours: whether the mother was licking the cub’s anogenital region, nursing the cub, grooming the cub, and holding the cub (i.e., whether the mother was supporting the cub on her body or not). We grouped any other behaviour involving the cub that is not described above into an “other maternal behaviour” category (e.g., olfactory investigation of cub, repositioning cub), which was also recorded using instantaneous sampling at one-minute intervals. The end of each one-minute sample interval was denoted by an audible signal from a watch. An observer recorded the presence or absence of the behaviours at the instant the signal sounded. The score obtained is a proportion of all sample points on which the behaviour was occurring^42^. We also noted the incidence of cub vocalisations using one-zero sampling at one-minute intervals^41^,^42^. For one-zero sampling, at the instant the signal sounded an observer recorded whether or not the cub had vocalised during the preceding one-minute interval. Table 2 provides descriptions of all the behavioural measures.

Twelve observers recorded behavioural data and the level of inter-observer reliability was calculated using an index of concordance^42^. Observers were considered reliable when the index of concordance with the first author was greater than 0.85 for each behaviour during an initial observation period. It took 10−20 hours of observation time for each observer to reach this level and data that did not meet this reliability criterion were not included in the analysis. After this level of inter-observer reliability was achieved, observers collected data alone. Observers watched the subjects from a position outside of the enclosures, usually at a distance of 1−5 meters from the subjects. Animal care staff monitored the subjects 24 hours per day throughout the study period, and thus the subjects were habituated to human presence.

### Statistical Analysis

Generalized linear mixed models (GLMMs) with a log link function and a Poisson probability distribution were used to examine the data. The log transformation and Poisson distribution should be used when the dependent variable represents a count of occurrences over time, as in the current study. Whether giant panda mothers had prior experience of raising offspring or not (experienced versus inexperienced) was entered as a fixed factor categorical variable in the GLMMs, and the dependent variables were the six behavioural measures (see Table 2). Because some of the giant panda mothers were represented more than once in the dataset we entered subject identity as a random factor in the GLMMs. Accordingly, the unit for statistical inference in the current study is the number of observation sessions for inexperienced (664 observations) and experienced mothers (1341 observations), not the total number of giant panda mothers (N = 8). This allowed us to capture daily variation in maternal care associated with the cub’s age. In addition, by entering the identity of each giant panda mother as a random factor in the analysis we also control for any potential variation associated with differences in body condition that may have existed between observation sessions and across individuals. The mean body weight for each of the giant panda mothers during the time the behavioural observations were conducted, the cub’s age, and whether mothers had twins or singletons were also entered as covariates in each of the GLMMs to control for these factors. The statistical analyses were conducted using IBM SPSS statistics version 20 (SPSS Inc, Chicago, IL, USA), significance levels were set at 0.05, and two-tailed statistics were used.

## Additional Information

**How to cite this article**: Snyder, R. J. *et al*. Giant Panda Maternal Care: A Test of the Experience Constraint Hypothesis. *Sci. Rep.* **6**, 27509; doi: 10.1038/srep27509 (2016).

## Figures and Tables

**Table 1:** Information about the identity, year data were collected, birth history, mean body weight, litter size, captive location, and hours of data collected on the giant panda mothers included in the study.

**Female giant panda**	**Year**	**Parity**	**Mean body weight (kg)**	**Litter size**	**Location**	^**#**^**Hours of data**
BingBing	2000	Multiparous	90	twins	CRBGPB	75.5
ChengCheng	1998	Multiparous	97	single	CRBGPB	68.5
	2000	Multiparous	100	twins	CRBGPB	61
ChengJi	2007	Primiparous	99	twins	CRBGPB	75.5
ErYatou	2007	Multiparous	93	twins	CRBGPB	72
LunLun	2006	Primiparous	103.5	single	Zoo Atlanta	101
	2008	Multiparous	99.4	single	Zoo Atlanta	77.5
MeiMei	1999	Primiparous	110	twins	CRBGPB	82
QingQing	2000	Multiparous	86	single	Chengdu Zoo	78.5
	2001	Multiparous	85	twins	CRBGPB	68
YaYa	1997	Primiparous	105	single	CRBGPB	73
	1999	Multiparous	107	twins		94
	2001	Multiparous	107	twins	CRBGPB	75.5
**TOTAL**						**1002**

**Table 2:** Descriptions of the behavioural measures that were recorded for captive giant panda mother-cub dyads in this study.

**Behaviour**	**Description**
Grooming cub	Mother licks any part of cub’s body, other than the anogenital area, and/or bites the cub lightly and repetitively, using incisors, anywhere on its body.
Nursing cub	Mother is alert or relaxed while cub suckles from nipples. This behaviour takes precedence over all other behaviours (e.g., licking cub anogenital).
Lick cub anogenital	Mother licks the cub’s anogenital area.
Holding cub	Mother uses any part of her body (paw, mouth, foreleg) to hold/support the cub on her body. At least 50% of the cub’s body must be supported on some part of the mother’s body.
Other maternal	Mother performs any other behaviour involving the cub that is not described above (e.g., olfactory investigation of cub, repositioning cub).
Cub vocal	Cub makes any type of vocalisation. May range from low-pitched throaty sound to high-pitched open-mouthed call.
